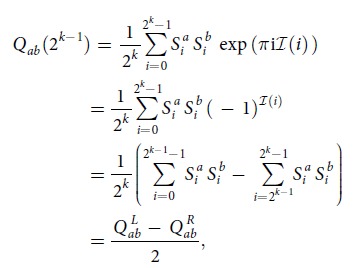# Erratum: Non-perturbative effects in spin glasses

**DOI:** 10.1038/srep12367

**Published:** 2015-12-18

**Authors:** Michele Castellana, Giorgio Parisi

Scientific Reports 5: Article number: 869710.1038/srep08697; published online: 03032015; updated: 12182015

This Article contains a typographical error in the Introduction section.

although the existence of a such a transition below the upper critical dimension is still under debate^16,17^.

should read:

although the existence of such a transition below the upper critical dimension is still under debate^16,17^.

In addition, there are errors in the following equations.

In Equation (14)


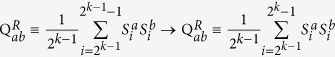


should read:


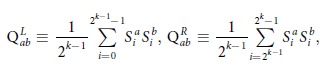


In Equation (15)


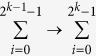


should read:


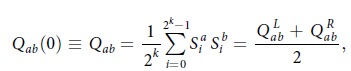


In Equation (16)


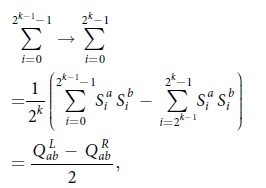


should read: